# ^68^Ga-Labeling: Laying the Foundation for an Anti-Radiolytic Formulation for NOTA-sdAb PET Tracers

**DOI:** 10.3390/ph14050448

**Published:** 2021-05-10

**Authors:** Henri Baudhuin, Julie Cousaert, Philippe Vanwolleghem, Geert Raes, Vicky Caveliers, Marleen Keyaerts, Tony Lahoutte, Catarina Xavier

**Affiliations:** 1 Department of Medical Imaging (MIMA), Vrije Universiteit Brussel, Laarbeeklaan 103, B-1090 Brussels, Belgium; philippe.vanwolleghem@ppms.be (P.V.); Vicky.Caveliers@uzbrussel.be (V.C.); Marleen.Keyaerts@uzbrussel.be (M.K.); Tony.Lahoutte@uzbrussel.be (T.L.); Catarina.Xavier@vub.be (C.X.); 2 Nuclear Medicine Department (NUCG), Universitair Ziekenhuis Brussel (UZ Brussel), Laarbeeklaan 101, B-1090 Brussels, Belgium; Julie.Cousaert@uzbrussel.be; 3 Unit of Cellular and Molecular Immunology (CMIM), Vrije Universiteit Brussel, Pleinlaan 2, B-1050 Brussels, Belgium; Geert.Raes@vub.be; 4 Myeloid Cell Immunology Laboratory (MCI), VIB Center for Inflammation Research, Pleinlaan 2, B-1050 Brussels, Belgium

**Keywords:** ^68^Ga, NOTA-sdAb, radiolysis, antioxidant, radioprotectant

## Abstract

During the preparation of [^68^Ga]Ga-NOTA-sdAb at high activity, degradation of the tracers was observed, impacting the radiochemical purity (RCP). Increasing starting activities in radiolabelings is often paired with increased degradation of the tracer due to the formation of free radical species, a process known as radiolysis. Radical scavengers and antioxidants can act as radioprotectant due to their fast interaction with formed radicals and can therefore reduce the degree of radiolysis. This study aims to optimize a formulation to prevent radiolysis during the labeling of NOTA derivatized single domain antibody (sdAbs) with ^68^Ga. Gentisic acid, ascorbic acid, ethanol and polyvinylpyrrolidone were tested individually or in combination to find an optimal mix able to prevent radiolysis without adversely influencing the radiochemical purity (RCP) or the functionality of the tracer. RCP and degree of radiolysis were assessed via thin layer chromatography and size exclusion chromatography for up to three hours after radiolabeling. Individually, the radioprotectants showed insufficient efficacy in reducing radiolysis when using high activities of ^68^Ga, while being limited in amount due to negative impact on radiolabeling of the tracer. A combination of 20% ethanol (VEtOH/VBuffer%) and 5 mg ascorbic acid proved successful in preventing radiolysis during labeling with starting activities up to 1–1.2 GBq of ^68^Ga, and is able to keep the tracer stable for up to at least 3 h after labeling at room temperature. The prevention of radiolysis by the combination of ethanol and ascorbic acid potentially allows radiolabeling compatibility of NOTA-sdAbs with all currently available ^68^Ge/^68^Ga generators. Additionally, a design is proposed to allow the incorporation of the radioprotectant in an ongoing diagnostic kit development for ^68^Ga labeling of NOTA-sdAbs.

## 1. Introduction

The success of molecular imaging relies on the availability of adequate radiopharmaceuticals, which are targeting vehicles labeled with a radionuclide to target a specific site in the body. Single-domain antibodies (sdAbs) have shown to be ideal targeting vehicles and have so been at the basis of several radiopharmaceuticals, both for imaging [1,2,3,4] and therapy [5,6]. SdAbs, usually derived from heavy-chain only antibodies [7], can easily be conjugated with a NOTA-chelator for ^68^Ga-labeling in their development as PET tracers [8,9]. Such sdAb-based PET tracers show a rapid and favorable biodistribution and can provide enough contrast in humans for imaging already at 60–90 min post injection [8]. Two [^68^Ga]Ga-NOTA-sdAb tracers are currently in clinical investigation as PET tracer: the [^68^Ga]Ga-NOTA-anti-HER2 tracer [8], for HER2 positive metastatic breast cancer diagnosis, including brain metastases, (EudraCT 2016-002164-13—NCT03924466; EudraCT 2015-002328-24—NCT03331601) and the [^68^Ga]Ga-NOTA-anti-MMR tracer [9], targeting the Macrophage Mannose Receptor (MMR), for detection and imaging of Tumor Associated Macrophages (TAM’s) (EudraCT 2017-001471-23—NCT04168528).

The correct preparation of these tracers, especially in clinical setting, is critical and could be facilitated by the development of a kit, such as it has been the case for ^68^Ga-PSMA (Illumet, Telix), ^68^Ga-DOTATATE (NETSPOT, AAA) and ^68^Ga-DOTATOC (SomaKit TOC, AAA). Not only can a kit simplify the labeling procedure by omitting a final purification and filtration step and by reducing the quality control (QC) to a minimal quality check of radiochemical purity, it can also facilitate transport and distribution of the precursor, in a convenient all-in-one package, comprising all additional (except the radionuclide) required solutions, compounds and/or accessories for a successful radiolabeling. Additionally, a kit has the advantage of having a standardized preparation procedure, while no specific GMP license is required for the preparation of a radiopharmaceutical according to a kit as it is no longer considered as an in-house preparation. For the purpose of developing such a kit, NOTA-sdAb precursors were previously lyophilized to improve their stability and increase the storage temperature from −20 °C to 2–8 °C. The formulation consists mainly of a mixture of sucrose and mannitol, which provides an elegant cake in the vial. However, degradation of the tracer was observed upon labeling at high activities. Degradation of compounds due to radioactivity, or radiolysis, is a well-known phenomenon for radiopharmaceuticals, one which also affects the [^68^Ga]Ga-NOTA-sdAb tracers. Radionuclides can, to a low extent, induce direct damage due to direct ionization of the radiopharmaceutical by the emitted ionizing radiation [10]. Indirectly, and much more prominent, the ionizing radiation (including γ-radiation) can induce damage by the formation of a variety of free radicals such as hydroxyl radicals (HO•), aqueous electron (eaq-), superoxide (O2•−) or other highly reactive species (H_2_O+, H_2_O− or H_2_O_2_), mainly by interaction with water molecules, abundantly present in aqueous solutions [10,11,12,13,14]. Such highly reactive compounds tend to degrade organic compounds, such as peptides, proteins, DNA-sequences etc. Especially protein-based targeting vehicles, such as antibodies or fragments thereof, are particularly sensitive to such radiolytic degradation or radiolysis, with consequently potential loss of immunoreactivity or binding capability [15,16,17]. This can render the radiotracer unusable and can cause increased radiation toxicity to certain organs when injected, as these degraded radioactive species might accumulate more easily in non-target organs [18]. Additionally, this can lead to reduced image quality, due to increased non-specific signal. The degree of radiolysis becomes an increasingly important factor with increasing activities, activity concentrations and specific activities [19,20,21,22]. Additionally, the degree of radiolysis is also influenced by the type of radiation emitted by the chosen radionuclide and the dose rates [12]. It is evident that radiolysis is an undesired occurrence, which can be prevented or diminished by adding antioxidants or scavenger molecules in the reaction mixture. Such molecules will interact with and neutralize the formed radicals, thereby protecting the targeting vehicle.

It has, in that regard, become common to develop anti-radiolytic formulations comprising a radioprotectant (RP) when carrying out a radiolabeling [12], whereby several factors should be taken into account. Firstly, the RP should not interact with or cause detrimental effects on the radiopharmaceutical or on the radiolabeling (in case the RP is added prior to radiolabeling). Secondly, depending on the formed radicals, one RP might be more efficient over another to protect a particular Active Pharmaceutical Ingredient (API). Proven efficacy of a RP for one radiopharmaceutical does not guarantee the same efficiency for another [23]. Thirdly, the radioprotectant should be fit for human use and should remain in acceptable amounts to be injected. A variety of molecules have so been investigated as potential RPs, including, but not limited to, gentisic acid (GA) [18,24], ascorbic acid (AA) [18,25,26], methionine [25], melatonine [27,28,29], ethanol (EtOH) [30,31], selenomethionine [25,32], and human serum albumin (HSA) [33].

Since radiolysis also occurs during ^68^Ga-labeling of NOTA-sdAbs, the aim is to develop a suitable anti-radiolytic formulation for these tracers. With the recent market approvals of several ^68^Ge/^68^Ga generators (E&Z—2014, IRE—2019 and ITG—2019), ^68^Ga is gaining important traction in nuclear medicine for radiopharmaceutical PET tracers [34]. These generators can deliver up to 1.3 GBq of effective activity (approximately 70% from the registered maximum activity of 1.85 GBq), when first used in clinical practice. To guarantee compatibility of [^68^Ga]Ga-NOTA-sdAbs with these generators, finding a suitable anti-radiolytic formulation is necessary. This is especially true for [^68^Ga]Ga-NOTA-sdAb in kit form, where the preparation of the final radiopharmaceutical should be as simple as possible, and where a final purification, intended to remove uncomplexed ^68^Ga and potential radiolytic product, is omitted.

In this study, GA, AA, EtOH and polyvinylpyrrolidone (PVP) K12 are investigated for their potential interference with ^68^Ga labeling, their radioprotective capabilities and their compatibility with the [^68^Ga]Ga-NOTA-anti-HER2 and [^68^Ga]Ga-NOTA-anti-MMR sdAb, to develop an optimized anti-radiolytic formulation, without adversely affecting the radiolabeling or the functionality of the radiotracer and which can be integrated in an ongoing kit development.

## 2. Results

Both NOTA-anti-HER2 and NOTA-anti-MMR precursors were used in the development of an anti-radiolytic formulation. However, due to fluctuation in availability of both precursors, some tests were performed only with one or the other. Critical confirmation experiments were carried out on both precursors, and on lyophilized precursor, if relevant.

### 2.1. Compatibility Testing of Different RPs with ^68^Ga Labeling

Each potential RP was tested individually as first assessment for their compatibility with ^68^Ga-labeling (Table 1). Different concentrations of GA and AA were tested, while PVP and ethanol were only tested at 50 mg/mL and 20% (V(_EtOH)_/V(_buffer)_%), respectively. The RPs were dissolved in 1M NaOAc pH5 radiolabeling buffer, while the ^68^Ga-activities were limited and ranged between 300 and 500 MBq to not induce radiolysis in any condition. The radiochemical purity (RCP) was analyzed 10 min and 3h (GA and AA only) post-labeling.

GA shows a decrease in RCP with increasing concentration, suggesting an interaction of GA with ^68^Ga ions, while AA shows high and comparable RCP with increasing concentrations, suggesting a good compatibility of AA with the ^68^Ga radiolabeling. PVP and ethanol were both tested at only one concentration and showed a high RCP after 10 min of incubation.

Based on the positive outcome of the AA compatibility test, we additionally assessed the potential of using AA as alternative buffer system to the acetate buffer. AA could be co-lyophilized and could allow for a direct elution of the ^68^Ga eluate into the lyophilized vial, ommitting the usage of a sodium acetate buffer. To this purpose, a freshly prepared 0.5 M ascorbic acid—sodium ascorbate pH 5 solution was tested for ^68^Ga radiolabeling and the RCP was analyzed 10 min post labeling (Supplemental Table S1). An end pH of approximately 4.5 was obtained, showing that the 0.5 M AA pH 5 (equivalent to 88 mg/mL AA) is a suitable buffer system. However, the RCP decreased significantly, suggesting that AA at such a concentration competes with the NOTA chelator for ^68^Ga complexation. Additional experiments were performed to verify the potential interference of AA with the ^68^Ga-labeling (Supplemental Table S2).

### 2.2. Ethanol

A more in-depth compatibility assessment was performed for EtOH to verify the potency of EtOH as RP and its impact on the protein and functionality and to assess a maximum tolerable amount.

In order to study the effect of ethanol on aggregation and precipitation of the unconjugated sdAbs, anti-MMR and anti-HER2 sdAb were exposed to different amounts of EtOH (0% up to 60% *v/v*%) in a 0.1M NaOAc buffer pH ~7. These samples were visually inspected and analyzed via SDS-PAGE 30 min after sample preparation or after storing the samples overnight in the fridge at 2–8 °C (Supplemental Figure S1).

Upon visual inspection no precipitation was observed in any of the fresh samples, but visible precipitation was observed for the anti-HER2 sdAb in the presence of 60% EtOH and for the anti-MMR sdAb starting at 50% EtOH in sample stored overnight. SDS-PAGE analysis showed no formation of aggregates nor signal at high molecular weight as only a single major band was detected between 10 and 15 kDa, corresponding with monomeric sdAb. It is possible that the precipiation and potential aggregation was reversed during sample preparation, which consists of a dilution with sample buffer and a heating step at 95 °C.

After this first evaluation, the effect of EtOH, up to 40%, during radiolabeling was studied (Table 2). This study was performed on lyophilized samples to also have an indication of compatibility with the existing lyophilization excipients, sucrose, mannitol and polysorbate 80. After radiolabeling the solutions were filtered (0.22 µm filter) to assess the formation of precipitate.

Upon filtration of the labeling solution without EtOH, about 3% of activity remains on the filter. It can be expected that a minimal residual amount of liquid remains on the filter due to the adhesion force of aqueous solutions. Carrying out a a radiolabeling with 20% EtOH in the buffer did not result in additional remaining activity on the filter. With 30% EtOH in the buffer, a slight increase in remaining activity (6.4% for the [^68^Ga]Ga-NOTA-anti-MMR and 4.9% for the [^68^Ga]Ga-anti-HER2) occurred, suggesting a minimal precipitation of the tracer, while at 40% EtOH more than 65% of the [^68^Ga]Ga-NOTA-anti-MMR tracer and more than 40% of the [^68^Ga]Ga-NOTA-anti-HER2 tracer remained on the filter, suggesting a high precipitation of the tracer. As such, the EtOH content was set to 20% *v*_(EtOH)_/*v*_(buffer)_% in the 1M NaOAc radiolabeling buffer (which results in 10% ethanol content upon radiolabeling) for further development and testing.

A first high activity test (>1 GBq) was performed to have an indication of the potency of 20% EtOH in the labeling buffer to reduce radiolysis (Table 3).

When performing a labeling in the presence of 20% EtOH, RCP was higher compared to the condition where no EtOH was used. Moreover, the peak corresponding to radiolysis compounds (peak 2 on iTLC) was lower at 10 min after labeling and only increased slightly 3 h after labeling (Table 3). These results show that EtOH is capable of reducing radiolysis, especially for long term stability, however, 20% EtOH in the labeling buffer is not sufficient to prevent radiolysis.

To investigate the effect of EtOH on the functionality of the precursors, the NOTA-sdAbs, exposed to 20% EtOH, were tested for affinity via Surface Plasmon Resonance (SPR) (Table 4).

The affinity (a measurement for strenght of interaction with the antigen) is represented as equilibrium dissociation constant K_D_ (k_off_ rate/k_on_ rate), where a lower K_D_ is correlated with a higher affinity and vice versa. No difference in affinity was observed either between the lyophilized and non-lyophilized NOTA-anti-MMR precursor, suggesting that 20% EtOH does not affect functionality even in the presence of the lyophilization excipients. Comparable results were obtained for the NOTA-anti-HER2 precursor, confirming the compatibility of these precursors with this ethanol content. The increased K_D_ of the NOTA-anti-HER2 exposed to 20% EtOH can be considered within the margin of error of the measurement rather than loss of affinity.

### 2.3. Effect of Combining Radioprotectants

The first high activity experiments showed that 20% EtOH as stand-alone was not potent enough to minimize radiolysis to acceptable levels. In order to decrease the radiolisys effect we evaluated the effect of combining EtOH with other RP.

Firstly, we evaluated the combination of EtOH with PVP K12 (Supplemental Table S3). The combination of 50 mg PVP K12 with 20% EtOH was not potent enough to prevent radiolysis (5% after 10 min and 12% after 3 h). Additionally, since the solubilizing properties of PVP could potentially counteract the precipitation that occurs at higher concentrations of EtOH, a labeling with 50 mg PVP K12 and 40% EtOH was also performed. Altough the degree of radiolysis reduced to acceptable levels (2% after 10 min and 3% after 3 h), it could be that precipitation of the radiolytic product still occurred and a false analysis of the product integrity is made. Surprisingly, the amount of unlabeled ^68^Ga increased (31%), which could be due to increased chelating capacity of PVP in presence of ethanol or due to reduced ^68^Ga complexation capability of precipitated precursor or a combination thereof. A third labeling was performed with 100 mg of PVP K12 without EtOH to assess the chelating capacity of PVP towards ^68^Ga and its potency as stand-alone RP. Despite the higher amount used, this labeling showed an improved labeling reaction as the amounty of uncomplexed ^68^Ga (7% after 10 min and 1% after 3 h) was lower, compared to any of the PVP/EtOH combination. However, the protection against radiolysis was minimal, as 13% of radiolysis occured after 10 min and 24% after 3 h.

Due to poor results of PVP as stand-alone RP and in combination with EtOH, additional studies were performed to assess the potential of combining 20% EtOH with AA and GA in different amounts (Supplemental Table S4). To overcome the interference of these compounds with ^68^Ga, resulting in lower RCP, the mass of NOTA-sdAb was increased from 100 µg to 200 µg per labeling condition. This increase in mass resulted in RCP > 99% after 10 min even at 5 mg/mL of AA or GA.

### 2.4. Design and Optimization of Anti-Radiolytic Formulation

In the first step towards a final formulation, the combination of 20% EtOH—5 mg/mL AA in buffer was tested in increasing radiolabeling volumes (2.2, 5, 7.5 and 10 mL total labeling volume). This was performed to verify the potential of using different ^68^Ga generators, which yield different elution volumes, such as Eckert & Ziegler’s GalliaPharm (0.1 N HCl—5 mL elution volume) or ITG’s ^68^Ga generator (0.05 N HCl—4 mL elution volume).

For each condition, three labelings were performed and tested for RCP 10 min and 3 h after radiolabeling (Table 5).

Increasing the total labeling volume to 7.5 and 10 mL resulted in a decreasing RCP after 10 min incubation. This is related to the increasing mass of AA in the reaction, since the buffer composition is 5 mg/mL AA, and the fact that NOTA-sdAb precursor is present is a lower concentration (due to the dilution factor). After three hours an RCP >99% is obtained, suggesting that AA is a weak chelating agent, that has an impact on the ^68^Ga-NOTA complexation rate.

In order to avoid that AA has an impact on RCP in the different reaction volume conditions, a fixed amount of 5 mg AA for each labeling volume was tested (Table 6). This resulted in an increase in RCP, reaching >99% even at 10 mL, the highest radiolabeling volume tested. The 20% EtOH—5 mg AA (fixed) formulation was also evaluated at high activities in both the 2.2 mL and 10 mL final radiolabeling volumes for both NOTA-sdAb, which showed that a RCP >95% could still be achieved with no radiolysis, even 3 h after labeling.

### 2.5. Osmolality and Dynamic Light Scattering Studies

The osmolality of different solutions, containing either no excipients, the lyohpilization excipients or a combination witht the radioprotectants, was analyzed to investigate the impact of different compounds on the osmolality, while mimicking the conditions as if the solution would be injected as final solution after radiolabeling. For each solution, the consistency and osmolality are presented in Table 7.

The reference solution, containing solely sodium acetate and precursor, already shows a relatively high osmolality of 811 mOsm/kg (a solution of 300 mOsm/kg is considered isotonic). The addition of the excipients for lyophilization in solution 1 has a minor impact on the osmolality, while ethanol in solution 2 greatly increases the osmolality to nearly 2500 mOsm/kg. Addition of AA in solution 3 further increases the osmolality slightly to nearly 2700 mOsm/kg. As expected, no major difference is observed between solution 3 in a 2.2 mL and 10 mL volume. This, however, confirms the strong influence of ethanol on the osmolality of the solutions. A 1:3 dilution of solution 3 with water for injection (WFI) was tested as well to verify if an osmolality of approximately 1000 mOsm/kg could be achieved, as this has been proposed as recommended upper limit by Wang et al. [35], while retaining a reasonable injection volume, as this would translate to a maximum total injection volume of 30 mL.

Particle size analysis was performed via Dynamic Light Scattering on solution 3 in a 2.2 mL labeling volume to analyze the distribution of particles in the solution. The solution was tested in triplicate. The mean hydrodynamic diameter (Dh) for each run is 0.78, 1.11 and 0.82 nm, respectively, resulting in an overall average of 0.90 nm with σ = 0.15 (Supplemental Figure S2). No particles above 3 nm Dh were measured, which suggests a clear and pure solution and no microprecipitation of any of the compounds nor aggregation of the tracer.

## 3. Discussion

In this study, we set out to develop an anti-radiolytic formulation to prevent radiolyis during and after preparation of [^68^Ga]Ga-NOTA-sdAbs. Four different potential radioprotectant candidates were investigated for this purpose based on their background in the literature.

- Gentisic acid has long been used as radiostabilizer, initially for ^99m^Tc labeled tracers [36,37]. GA is a strong anti-oxidant and free-radical scavenger [24], with a low toxicity profile. Preliminary studies have shown even potential health benefits regarding cardioprotection and antitumor activities [38]. GA, however, showed interference with the labeling at a concentration of 1 mg/mL in the radiolabeling buffer when using 100 µg of NOTA-sdAb and even more so with 5 mg/mL.
- Ascorbic acid, also known as Vitamin C, is a well-known and potent natural antioxidant and has the ability to protect other molecules (e.g., DNA, proteins) from highly reactive or oxidizing agents, such as free radicals. Hence, Vitamin C has proven to be an attractive candidate as additive during reactions with radioactive compounds [26]. Additionally, ascorbic acid has also been proposed as alternative buffer system for metalloradiopharmaceuticals [26,39]. The use of an AA buffer system did not seem to be compatible with ^68^Ga, as the labeling of the NOTA-sdAbs was severely impacted when using a 0.5 M AA buffer pH 5. This shows that AA, as well as GA, has chelating capacity towards ^68^Ga-ions.
- Ethanol has since long been used as co-solvent in the production of [^18^F]FDG for anti-radiolytic purposes. Recently, it has also been integrated in automated ^68^Ga radiolabeling synthesis [40,41]. The maximum tolerable amount for the NOTA-sdAbs was 20%, as precipitation occurs at higher amounts. At this amount, EtOH showed some efficiency as radioprotectant during the ^68^Ga radiolabeling but was not able to reduce radiolysis to acceptable levels. Exposing the NOTA-sdAbs to 20% EtOH did not affect the affinity. Interestingly, EtOH has shown the ability to significantly improve the complexation reaction of radiometals, including ^68^Ga [33,40,42,43].
- PVP, also known as Povidone, is obtained via polymerization of monomer N-vinylpyrrolidon. Different chain-lengths can be polymerized with different molecular weights which, consequently, have different viscosities and physical properties (denoted with a K-value). An unusual property of PVP is its good solubility in both aqueous and organic solvents, facilitating or broadening the usage of PVP [44]. Although little information is available, a granted US patent (5961955) describes the usage of PVP as excipient in radiopharmaceutical preparations to reduce radiolysis. In the ^68^Ga studies presented here, addition of 100 mg PVP K12 during radiolabeling showed little efficiency to reduce radiolysis. Additionally, the combination of PVP and ethanol has an impact on the ^68^Ga complexation reaction most likely due to increased chelating capacity of PVP in presence of EtOH, a phenomenon studied and shown by Liu et al. [45].

Finding a suitable radioprotectant or a combination thereof to prevent radiolysis during ^68^Ga-radiolabeling was quite challenging. The main causes of difficulty were the undesired effects of radioprotectants on the tracer or on the radiolabeling which limit the usable amount of a particular RP. Therefore, a combination of different RP’s had to be found to minimize/prevent their negative effects and maximize their anti-radiolitic effect. Although working with the DOTA chelator, Velikyan et al. stumbled upon the same effect of decreased RCP upon addition of GA and AA during ^68^Ga radiolabeling in the development of an anti-radiolytic formulation for a glucagon like peptide-1 analogue, which resulted in a mixture of 10% EtOH in the reaction volume and 3.5 mmol/L of GA and AA [41]. To ensure an optimal labeling efficiency and to overcome the interference of GA and AA with the labeling, the mass of NOTA-sdAb was increased to 200 µg. Additionally, due to the described benefits of increased complexation rate with EtOH and inherent stability of this compound, EtOH was chosen as first excipient in a potential combination. At low activity, the combination of EtOH with GA and AA provided RCP >99%. The combination of 20% EtOH and AA at 5 mg/mL in the buffer was further tested in varying radiolabeling volumes to assess the potential compatibility with different ^68^Ga generators. Here, we found that at higher labeling volumes, the labeling efficiency decreased, a combination of the higher labeling volume and the presence of a higher mass of AA. As such, we opted to fix the amount of AA to 5 mg irrespective of the buffer volume used. This resulted in an RCP >99% even with the largest labeling volume of 10 mL. The combination of 20%EtOH—5 mg AA was tested at high activity for both NOTA-sdAb precursors in a small (2.2 mL) and large (10 mL) labeling volume, which yielded an RCP >98% after 10 min and >99% after 3 h, showing an efficient protection against radiolysis up to 3 h after labeling. A benefit of combining different RPs, is a more broad protection towards different types of radicals that might be formed, as each RP will provide optimal protection against a particular radical. EtOH is a well-known strong hydroxil radical scavenger [46], while ascorbic acid can provide protection against different radicals [47].

With the development of a cold kit for ^68^Ga labeling of NOTA-sdAbs, the anti-radiolytic compounds should be incorporated in this design. The kit consists of one vial containing lyophilized NOTA-sdAb precursor and one vial of 1 M NaOAc buffer pH 5. The ethanol can easily be added to the buffer vial yielding a 1 M NaOAc/20% EtOH pH 5 buffer solution, while 5 mg of AA can be added to the current existing lyophilization formulation. This should allow a long-term stability of the AA, as it is highly unstable in solution.

Regarding patient concern and safety, two points can be adressed. Firstly, the osmolality of the final solution is relatively high. However, no upper limit for osmolality is specified in the European Pharmacopeia. To keep discomfort to a minimum upon injection, it is recommended to remain below 1000 mOsm/kg for intravenous injection of small volumes (≤100 mL) in adults [35]. A three times dilution of the final solution with injection water resulted in an osmolality of approximately 1100 mOsm/kg, therefore an osmolality <1000 mOsm/kg could easily be reached with a 3.5 to 4 times dilution. Secondly, the presence of ethanol in the final solution could be a concern. However, the amount is relatively limited. The maximum amount of ethanol that would potentially be injected using the kit is only 1 mL (10% of 10 mL), resulting in a 0.16 g/L blood concentration, considering a relatively low total blood volume of 5L. This is well below the 0.5 g/L limit set for drivers in Belgium. Additionally, it should be noted that the injected activity for imaging is typically relatively low (approximately 185 MBq for ^68^Ga[Ga]-NOTA-sdAbs) compared to the starting activity (which can go up to 1.3 GBq for the current market approved generators). As such, it is unlikely that the total radiolabeling solution will be injected in one patient, rather the volume corresponding to approximately 185 MBq will be retrieved in a syringe and only this will be injected in a patient.

Despite that only 185 MBq is sufficient for imaging, and that the kit is being developped as a single-patient product, being able to radiolabel at higher activities would be an advantage as this allows shipment of the final radiolabeled product. ^68^Ga has a relatively low half-life of 68 min, but with starting activities of approximately 1.3 GBq or up to 4 GBq in the future, it is feasible to have centralized productions and shipment to clinical centers, as it is often the case for fluor-18 labeled compounds. Especially in America the concept of centralized productions is well-established and is being used for ^68^Ga compounds as well. With this in mind, it is also crucial that the final radiolabeled product is stable for several hours after labeling. Moreover, even though we intend to develop the kit as a single-patient product, the ability to be able to use high activities for labeling, leaves open the option for also offering a kit that can be used for multiple patients. Based on our results that with 100 µg of lyophilized NOTA-sdAb precursor, even at lower activities, an RCP of at least 95% could not always be achieved in combination with some radioprotectants, we currently foresee that even in case of a kit as a single-patient product, a mass of 200 µg of lyophilized NOTA-sdAb will be inlcuded in the kit to always guarantee an RCP >95% with high activity in different labeling volumes. Radiopharmacies which manufacture the final product for local use can adjust the starting activity, so that the mass corresponding to 185 MBq remains within a specified range. In the current clinical trials 100 µg is the upper limit for the mass that can be injected into patients. The injected mass can vary depending on the specific activity and in practice typically between 50 and 75 µg is currently injected. While no lower limit has been set, the upper limit might be increased to 200 µg for the kit, the total mass that would be present, as to allow a complete injection of the final solution and to maximize the shipping radius.

Lower amounts of ethanol (e.g., 0%, 5%, 10% or 15%) in combination with 5 mg AA were not tested at high activity. Such combinations might prove adequate as well in preventing radiolysis, with the additional advantage of a lower osmolality, improving the injectability of the final solution. This refinement of the formulation still remains to be investigated and was not performed yet due to limited access to a generator with relevant activity to perform this study. Additionally, with the development of more powerful, 4 GBq ^68^Ga-generators, which provide up to double or even more starting activities compared to the 1.85 GBq generators, the anti-radiolytic formulation will have to be stress-tested with such activities, and adjusted accordingly if necessary, to allow compatibility of a NOTA-sdAb [48] labeling kit with these next generation generators.

## 4. Material and Methods

All commercially obtained chemicals were of analytic grade. The recombinant anti-HER2 (molecular weight = 12,628 Da) and anti-MMR sdAb-proteins (molecular weight = 12,678 Da) were produced without terminal tags by the VIB Protein Service Facility in Pichia pastoris and were formulated in PBS during the final batch purification. p-SCN-Bn-NOTA was purchased from Macrocyclics (Macrocyclics, Inc., Plano, TX, USA). ^68^Ga was obtained from a ^68^Ge/^68^Ga Galli Eo^TM^ generator (IRE, Belgium). High purity water (TraceSELECT™, for trace analysis, Riedel-de Haën, Honeywell Research Chemicals, Seelze, Germany) and ethanol (Ethanol EMSURE^®^, Ph..Eur., Merck, Darmstadt, Germany) were used in the preparation of any buffer or solution. High grade ascorbic acid (99.7–100.5%, puriss. p.a., Ph.Eur., Sigma-Aldrich, St. Louis 63103, MO, USA), gentisic acid (99%, Acros Organics, part of Thermo Fisher Scientific, Geel, Belgium), Polyvinylpyrrolidone (average MW 10,000 g/mol, Sigma-Aldrich, St. Louis, MO, USA) sucrose (≥99.5%, Sigma-Aldrich, St. Louis, MO, USA), D-mannitol (≥98%, Sigma-Aldrich, St. Louis, MO, USA) and polysorbate 80 (Ph.Eur., Aca Pharma, Nazareth, Belgium) were used in the respective buffer/solution preparation.

### 4.1. Conjugation of p-SCN-Bn-NOTA to sdAb Protein

SdAb proteins (Anti-HER2: 3–13mg, 0.24–1.03 μmol; Anti-MMR: 10–16 mg, 0.79–1.26 μmol) were buffer-exchanged to 0.5M sodium carbonate/0.15M NaCl buffer (Sodium carbonate anhydrous—Sodium hydrogen carbonate—Sodium Chloride, VWR Chemicals, Leuven, Belgium), pH 8.8–8.9, using PD-10 size exclusion disposable columns (GE Healthcare, Buckinghamshire, UK). Protein solution (2.2–2.4 mg/mL) was added to a twenty-fold (anti-HER2 sdAb) or thirty-fold (anti-MMR sdAb) molar excess p-SCN-Bn-NOTA. After 2 h incubation at room temperature (RT), the NOTA-sdAb protein solution was concentrated, if necessary, with Vivaspin 2 concentrator (MW cut-off 5kDa) (Sartorius Stedim Lab, Stonehouse, UK) and loaded on a SEC column. The collected fractions containing the monomeric NOTA-sdAb protein were pooled and the solution was passed through a 0.22 μm–13 mm filter (Millex, Merck Millipore, Tullagreen Carrigtwohill County Cork, Ireland). The protein concentration is determined by UV absorption at 280 nm (NOTA-anti-HER2 sdAb: ε = 49,690 M^−1^·cm^−1^, MW = 13,310 g/mol; NOTA-anti-MMR sdAb: ε = 40,660 M^−1^·cm^−1^, MW = 13,130 g/mol).

### 4.2. Preparation of [^68^Ga]Ga-NOTA-sdAb

The NOTA-sdAb precursor sample (100 µg or 200 µg, as specified) was first diluted or reconstituted with 1.1 mL of the respective 1 M NaOAc buffer (Sodium acetate trihydrate, ≥99.5%, puriss. p.a., Ph.Eur., Sigma-Aldrich Chemie, Steinhelm, Germany—Acetic acid, ≥99.8%, puriss. p.a., Ph.Eur., Sigma-Aldrich Chemie, Steinhelm, Germany) pH 5, after which the full ^68^Ga eluate (1–1.1 mL) was added. The sample was incubated for 10 min at RT, filtered, where specified, and then analyzed for radiochemical purity by iTLC and SEC (SEC: [^68^Ga]Ga-NOTA-sdAb R_t_ = 4.6 min, σ = 0.10 min; ^68^Ga-citrate R_t_ = 7.6 min, σ = 0.34 min; Radiolytic product R_t_ = 8.3 min, σ = 0.25 min; iTLC-SG: [^68^Ga]Ga-NOTA-sdAb R_f_ = 0.05 σ = 0.01, Radiolytic product R_f_ = 0.72, σ = 0.08 ^68^Ga-citrate R_f_ = 1.13, σ = 0.06). Where specified, after 3h, a second QC via iTLC and SEC was performed. Examples of chromatographic analysis are provided in Supplemental Figure S3.

In some studies, a higher radiolabeling volume is tested. In these cases, the ^68^Ga eluate was further diluted accordingly with 0.1 M HCl (Hydrochloric acid, ≥37% puriss. p.a., Ph.Eur., Sigma-Aldrich Chemie, Steinhelm, Germany) to retain a 1:1 ratio of buffer to eluate.

In the study to test the effiency of EtOH as RP, the [^68^Ga]Ga-NOTA-sdAb was prepared using a 1M NaOAc/20% EtOH radiolabeling buffer.

In the RP combination studies, the corresponding amount of PVP, AA or GA were weighed and added during the preparation of a 1 M NaOAc/20% EtOH radiolabeling buffer to obtain the desired concentration (50 or 100 mg/mL for PVP and 1 or 5 mg/mL for AA/GA).

In the final combination study of EtOH and AA, a fixed amount of 5 mg AA was weighed and dissolved in the corresponding volume (1.1 mL, 2.5 mL, 3.5 mL or 5 mL) of 1 M NaOAc/20% EtOH radiolabeling buffer. The solution was then stored at 2–8 °C in an interval between 20 and 28 h before carrying out a radiolabeling.

### 4.3. Chromatographic Analysis

Size exclusion chromatography (SEC) purification of NOTA-sdAb was conducted on an NGC Chromatography system (Bio-Rad Laboratories, USA) using a Superdex 30 pg HiLoad 16/600 column (GE Healthcare Bio-Sciences AB, Uppsala, Sweden) at a flow rate of 1 mL/min flow rate (mass > 6 mg) or a Superdex Peptide 10/300GL column (GE Healthcare Bio-Sciences AB, Uppsala, Sweden) at a flow rate of 0.5 mL/min (mass < 6 mg) and 0.1 M sodium acetate pH 7 (Sodium acetate trihydrate, ≥99.5%, puriss. p.a., Ph.Eur., Sigma-Aldrich Chemie, Steinhelm, Germany) as mobile phase. The latter was also used for quality control of NOTA-sdAbs.

Quality control (QC) of [^68^Ga]Ga-NOTA-sdAbs was performed on a Hitachi Chromaster Chromatography system (VWR, Leuven, Belgium) using SEC on a custom size Superdex 30 Increase 5/150 column (GE Healthcare Bio-Sciences AB, Uppsala, Sweden) at a flow rate of 0.3 mL/min flow rate and 0.02 M PBS/0.28M NaCl pH 7.4 (PBS Tablets, Merck, Darmstadt, Germany) as mobile phase. QC was also assayed with binderless glass microfiber paper that was impregnated with silica gel (instant thin layer chromatography, iTLC-SG) (Agilent Technologies, Diegem, Belgium) with 0.1 M sodium citrate pH 5 (Citric acid, trisodium salt, dihydrate—Citric acid monohydrate, Acros Organics, part of Thermo Fisher Scientific, Geel, Belgium) as mobile phase. The iTLC strips were measured via a miniGita Single TLC-scanner (Elysia-Raytest, Angleur, Belgium).

### 4.4. pH

The pH of solution was measured with a pH electrode Blueline 14 on a Lab 855 digital pH meter (SI Analytics, Mainz, Germany). Measurement of radiolabeling solutions was measured after decay (typically the next day).

### 4.5. Surface Plasmon Resonance

Surface Plasmon Resonance (SPR) was performed on a Biacore T200 (GE Healthcare) system as described previously [3,4]. Briefly, a CM5 chip was coated with either recombinant HER2Fc or recombinant hMMR via 1-ethyl-3-(-3-dimethylaminopropyl)carbodiimide (EDC) and N-hydroxysuccinimide (NHS) chemistry. The affinity was determined by flowing different concentrations of precursor over the immobilized protein. The obtained curves were fitted with a 1:1 sdAb:antigen binding model to calculate the binding parameters. A reference sample containing anti-HER2-(HIS)_6_ or anti-MMR-(HIS)_6_ sdAb, stored at −20 °C, was added during each run.

To assess the effect of EtOH on the affinity of NOTA-sdAbs, the NOTA-sdAbs were transferred to a 20% EtOH/0.1M NaOAc solution, placed overnight in the fridge and then stored at −20 °C for several weeks until SPR measurement.

### 4.6. SDS-PAGE

SDS-PAGE was performed on NOVEX Wedgewell 16% 10-well gel (Thermo Fischer Scientific, Carlsbad, CA, USA), where 10 and 2 µg of NOTA-sdAb was loaded in both reducing and non-reducing conditions. The gel was run at 80 V for 10 min, then at 150 V for 65, after which a Coomassie Blue staining was performed for detection. Gels were visualized with the Amersham 680 RGB Imager (GE Healthcare Bio-Sciences AB, Uppsala, Sweden) and analyzed via the GE ImageQuant TL 1D v 8.2.0 analysis software.

### 4.7. Osmolality

The osmolality of the formulations was measured using an Advanced^®^ Micro-Osmometer (Model 3300, Advanced Instruments Inc., Norwood, MA, USA) based upon the freezing point depression method. Calibration of the device was performed using Clinitrol™ 290 reference solution (Advanced Instruments Inc., Norwood, MA, USA). As the osmolality of some formulations was higher than the upper range value of 2000 mOsm/kg, all measured samples were diluted (1:1) with milliQ water and the result was multiplied by two. The measurements were conducted in triplicate (on 20-µL aliquots) and mean values were reported.

Following conditions were analyzed:

- Reference: 0.5 M NaOAc pH 5 buffer
- Basic condition: 0.5 M NaOAc pH 5 buffer + excipients lyophilization
- Intermediate condition: 0.5 M NaOAc/10% Ethanol pH 5 buffer + excipients lyophilization
- Final condition: 0.5 M NaOAc/10% Ethanol pH 5 buffer + excipients lyophilization + 5 mg VitC

Additionally, the final condition was tested in a concentrated and diluted form, simulating the 2.2 and 10 mL radiolabeling volume.

### 4.8. Particle Size Analysis

Dynamic Light Scattering (DLS) was applied to evaluate the presence of particles in the formulations. Measurements were conducted in triplicate at 25 °C using a Zetasizer Nano ZS apparatus (Malvern Instruments Ltd., Mavern, UK) with attenuator index 11, i.e., 100% transmission of the light through the sample.

## 5. Conclusions

A formulation, preventing radiolysis of the tracer during ^68^Ga-radiolabeling, has succesfully been developped. The additional excipients, ethanol and ascorbic acid, showed strong protection against radiolysis at the highest activity available from current commercially available ^68^Ga generators. The formulation provides protection for up to 3 h after radiolabeling, while these anti-radiolytic excipients can easily be integrated in a kit for ^68^Ga-labeling of NOTA-sdAbs. All the used components (ethanol, ascorbic acid, sodium acetate, sucrose, mannitol and polysorbate 80) are well-known EMA/FDA approved excipients, frequently used in a variety of pharmaceutical drugs and the amount of each component in the final product would be considered below their maximum acceptable limit for patient injection.

## 6. Patents

Vrije Universiteit Brussel submitted patent applications comprising concepts depicted in this manuscript.

## Figures and Tables

**Table 1:** Compatibility test of different radioprotectants for the 68Ga labeling of NOTA-sdAb.

Compound	RP	Concentration (mg/mL or %)	Method	RCP (%)	
NOTA-anti-HER2				10 min	3 h
	GA	0	iTLC	94	94
			SEC	93	91
		1	iTLC	87	89
			SEC	89	87
		5	iTLC	46	83
			SEC	50	84
	AA	0	iTLC	98	97
			SEC	98	96
		1	iTLC	97	94
			SEC	98	99
		5	iTLC	96	98
			SEC	97	98
	PVP K12	50	iTLC	96	/
	EtOH	0%	iTLC	99	/
		20%	iTLC	99	/

RP = Radioprotectant; RCP = Radiochemical Purity; iTLC = instant Thin Layer Chromatography; SEC = Size Exclusion Chromatography; Radioactivity ranged between 300 and 500 MBq; Mass precursor used per labeling = 100 µg; GA = gentisic acid; AA = ascorbic acid; PVP K12 = polyvynilpyrolidone K12; EtOH = ethanol; % EtOH = the percentage in the labeling buffer, before addition of ^68^Ga eluate.

**Table 2:** Effect of EtOH on ^68^Ga labeling of lyophilized NOTA-sdAb.

Compound	EtOH (%)	Activity (MBq)	Activity Filter (%) *	Method	RCP (%)	pH
NOTA-anti-MMR Lyo					10 min	
	0	549.0, σ = 19.6	3.0, σ = 0.2	iTLC	95.9, σ = 0.7	4.65, σ = 0.00
	20	542.3, σ = 9.1	3.3, σ = 0.3	iTLC	92.5, σ = 4.5	4.52, σ = 0.02
	30	522.0, σ = 15.3	6.4, σ = 0.9	iTLC	82.9, σ = 2.4	4.55, σ = 0.00
	40	536.7, σ = 14.3	66.9, σ = 26.1	iTLC	89.5, σ = 2.4	4.41 **
NOTA-anti-HER2 Lyo						
	0	498.0, σ = 13.0	2.9, σ = 0.4	iTLC	94.8, σ = 5.4	4.66, σ = 0.04
	20	483.7, σ = 2.6	2.4, σ = 0.7	iTLC	94.5, σ = 4.8	4.56, σ = 0.05
	30	476.0, σ = 14.3	4.9, σ = 0.4	iTLC	93.6, σ = 5.0	4.52, σ = 0.01
	40	481.3, σ = 15.1	42.5, σ = 3.5	iTLC	85.2, σ = 7.1	4.44, σ = 0.05

The experiment was repeated in triplicate for each condition; RCP = Radiochemical Purity; iTLC = instant Thin Layer Chromatography; Mass precursor used per labeling = 100 µg; % EtOH = % ethanol in the labeling buffer, before addition of ^68^Ga eluate; * The remaining activity on filters is presented as % compared to the initial activity in the vial minus the remaining activity in the vial after uptake of the solution, all decay corrected to timepoint of activity measurement of the solution after 10 min of incubation. ** Only two measurements.

**Table 3:** Effect of EtOH on radiolysis during high activity 68Ga labeling of lyophilized NOTA-sdAb.

Compound	EtOH (%)	Activity (GBq)	Method	QC/iTLC (%)						pH
				10 min			3 h			
NOTA-anti-MMR Lyo				1	2	3	1	2	3	
	0	1.06	iTLC	69	17	14	65	26	9	4.82
	20	1.17	iTLC	80	13	7	82	16	2	4.59

The iTLC strips were scanned and three main peaks were observed. 1 = Rf = 0 [^68^Ga]Ga-NOTA-sdAb; 2 = Rf = 0.7 radiolysis compound; 3 = Rf = 1 unlabeled ^68^Ga (^68^Ga-citrate); RCP = Radiochemical Purity; iTLC = instant Thin Layer Chromatography; Mass precursor used per labeling = 100 µg; % EtOH = % ethanol in the labeling buffer, before addition of ^68^Ga eluate.

**Table 4:** Effect of pre-incubation of NOTA-sdAbs with EtOH on precursor affinity, as measured via SPR.

Compound	EtOH (%)	K_D_ (nM)
NOTA-anti-MMR Lyo	20	1.6
NOTA-anti-MMR Lyo	0	1.2, σ = 0.3
NOTA-anti-MMR	20	1.6
NOTA-anti-MMR	0	1.2, σ = 0.2
anti-MMR Reference	0	1.2, σ = 0.3
NOTA-anti-HER2 Lyo	20	4.3
NOTA-anti-HER2 Lyo	0	4.7, σ = 1.1
NOTA-anti-HER2	20	6.1
NOTA-anti-HER2	0	4.8, σ = 1.6
Anti-HER2 Reference	0	3.7, σ = 0.7

**Table 5:** Exploratory study of combining 20%EtOH with 5 mg/mL AA in increasing radiolabeling volumes.

Compound	Volume (mL) *	Activity (MBq)	Activity Filter (%) **	Method	RCP (%)		pH
NOTA-anti-MMR					10 min	3 h	
	2.2	447.3, σ = 6.9	1.5, σ = 0.1	iTLC	99.8, σ = 0.1	99.8, σ = 0.1	4.50, σ = 0.03
				SEC	99.5, σ = 0.2	99.6, σ = 0.3	
	5	437.3, σ = 12.4	2.2, σ = 0.5	iTLC	97.3, σ = 1.5	98.9, σ = 0.5	4.40, σ = 0.04
				SEC	99.2, σ = 0.4	99.5, σ = 0.4	
	7.5	461.0, σ = 1.6	1.3, σ = 0.1	iTLC	91.4, σ = 6.4	98.7, σ = 1.1	4.51, σ = 0.18
				SEC	93.9 ***	99.4, σ = 0.2	
	10	467.3, σ = 12.7	1.0, σ = 0.1	iTLC	84.0, σ = 3.8	99.2, σ = 0.2	4.52, σ = 0.02
				SEC	93.3, σ = 1.3	99.4, σ = 0.2	

RCP = Radiochemical Purity; iTLC = instant Thin Layer Chromatography; SEC = Size Exclusion Chromatography; Mass precursor used per labeling = 200 µg; * total volume of reaction = 1:1 ratio of buffer/^68^Ga eluate; ** The remaining activity on filters is presented as % compared to the initial activity in the vial minus the remaining activity in the vial after uptake of the solution, all decay corrected to timepoint of activity measurement of the solution after 10 min of incubation. *** Only two measurements.

**Table 6:** Exploratory and confirmation study of combining 20%EtOH with 5 mg AA (fixed) in increasing radiolabeling volumes.

Compound	Volume (mL)	Activity (MBq)	Activity Filter (%) *	Method	RCP (%)		pH
NOTA-anti-MMR					10 min	3 h	
	2.2	536.7, σ = 16.1	1.7, σ = 0.0	iTLC	99.7, σ = 0.5	98.5, σ = 1.7	4.49, σ = 0.02
				SEC	99.6, σ = 0.1	99.7, σ = 0.2	
	5	372.0, σ = 8.8	2.1, σ = 0.3	iTLC	99.5, σ = 0.3	99.8, σ = 0.3	4.52, σ = 0.04
				SEC	99.0, σ = 0.4	99.1, σ = 0.2	
	7.5	443.7, σ = 2.4	1.7, σ = 0.3	iTLC	99.7, σ = 0.2	99.5, σ = 0.3	4.79, σ = 0.00
				SEC	99.4, σ = 0.2	99.3, σ = 0.3	
	10	447.0, σ = 7.8	1.6, σ = 0.2	iTLC	99.6, σ = 0.4	99.8, σ = 0.4	4.71, σ = 0.13
				SEC	99.1, σ = 0.2	99.2, σ = 1.2	
	2.2	1237.0, σ = 31.8	2.9, σ = 1.6	iTLC	99.8, σ = 0.2	99.9, σ = 0.1	4.42, σ = 0.06
				SEC	99.3, σ = 0.1	99.6, σ = 0.2	
	10	1174.7, σ = 18.6	1.6, σ = 0.6	iTLC	98.4, σ = 0.6	99.7, σ = 0.2	4.44, σ = 0.00
				SEC	98.5, σ = 0.2	99.7, σ = 0.2	
NOTA-anti-HER2							
	2.2	1060.3, σ = 29.0	1.8, σ = 0.4	iTLC	99.8, σ = 0.1	99.8, σ = 0.1	4.56, σ = 0.02
				SEC	99.2, σ = 0.2	99.4, σ = 0.5	
	10	1075.0, σ = 27.8	1.7, σ = 0.1	iTLC	98.5, σ = 0.4	99.6, σ = 0.4	4.55, σ = 0.12
				SEC	98.5, σ = 0.2	99.5, σ = 0.1	

RCP = Radiochemical Purity; iTLC = instant Thin Layer Chromatography; SEC = Size Exclusion Chromatography; Mass precursor used per labeling = 200 µg; * The remaining activity on filters is presented as % compared to the initial activity in the vial minus the remaining activity in the vial after uptake of the solution, all decay corrected to timepoint of activity measurement of the solution after 10 min of incubation.

**Table 7:** Osmolality of different conditions.

Sample	Volume (mL)	NOTA-sdAb (mg/mL)	Buffer Final Concentration	Kit Excipients *	EtOH (%)	AA (mg/mL)	Osmolality (mOsm/kg) ½ Diluted	Osmolality (mOsm/kg)
Reference	2.2	0.09	0.5 M NaOAc pH 5	/	/	/	405, σ = 3.8	811, σ = 7.6
Solution 1	2.2	0.09	0.5 M NaOAc pH 5	Yes	/	/	460, σ = 3.5	921, σ = 7.0
Solution 2	2.2	0.09	0.5 M NaOAc pH 5	Yes	10%	/	1248, σ = 2.9	2495, σ = 5.8
Solution 3	2.2	0.09	0.5 M NaOAc pH 5	Yes	10%	2.27	1349, σ = 13.5	2698, σ = 27.1
	10	0.02	0.5 M NaOAc pH 5	Yes	10%	0.5	1343, σ = 10.1	2685, σ = 20.2
Solution 3 (1:3 dilution with WFI)	30	0.007	0.017 M NaoAc pH 5	Yes	3.33%	0.17	/	1088, σ = 5.44

* kit excipients: sucrose, mannitol and polysorbate 80; AA = ascorbic acid.

## Data Availability

The raw data and processed data required to reproduce these findings are available to download from https://vub.sharepoint.com/:f:/r/teams/ORG-ICMI/External%20Share/Shared%20Documents/2021_Baudhuin%20et%20al_Radioprotectant?csf=1&web=1&e=0hGNrF (accessed on 10 May 2021). Access can be granted upon request.

## Supplementary Materials

The following are available online at https://www.mdpi.com/article/10.3390/ph14050448/s1. Table S1: Preparation of [68Ga]Ga-NOTA-sdAb using ascorbic acid buffer system; Table S2: Verification study AA; Table S3 Preparation of [68Ga]Ga-NOTA-sdAb in combination with EtOH and PVP; Table S4: Preparation of [68Ga]Ga-NOTA-sdAb in combination with EtOH and AA or GA. Figure S1: Effect of EtOH on protein aggregation via SDS-PAGE. Protein were exposed to an increasing amount of EtOH, from 0–60 from left to right. (A) Analysis of anti-HER2 protein exposed to EtOH and analyzed withing 30 min, (B) Analysis of anti-HER2 exposed to EtOH and incubated overnight at 2–8°, (C) Analysis of anti-MMR protein exposed to EtOH and analyzed withing 30 min, (D) Analysis of anti-MMR exposed to EtOH and incubated overnight at 2–8°; Figure S2: Graphical representation of the distribution of sized particles in the final concentrated formulation. Panel 2A shows that the majority of particles has a maximum hydrodynamic diameter of 3 nm. Panel 2B shows the complete measurement spectrum and confirms that no other, larger, particle sizes are present in the solution; Figure S3: Chromatographic analysis of 68Ga[Ga]NOTA-sdAb, RCP and radiolysis. Panel A shows SEC analysis of 68Ga[Ga]-NOTA-sdAb (Rt: 4.65 min) and non-incorporated 68Ga[Ga]citrate (Rt: 7.40 min), while Panel B shows 68Ga[Ga]-NOTA-sdAb (Rt: 4.60 min) with radiolytic degradation (Rt: 7.90 min—8.57 min). Panel C and D show the corresponding iTLC analysis, where Panel C shows 68Ga[Ga]-NOTA-sdAb (Rf: 0.04 (28.7 mm)) and non-incorporated 68Ga[Ga]citrate (Rf: 0.98 (107.8 mm)), while Panel D shows 68Ga[Ga]-NOTA-sdAb (Rf: 0.07 (30.0 mm)) and radiolytic degration (Rf: 0.73 (87.1 mm)).
