# Supplementary material for: 68Ga-Labeling: Laying the Foundation for an Anti-Radiolytic Formulation for NOTA-sdAb PET Tracers
Source: Pharmaceuticals (Basel). 2021 May 10;14(5):448. doi: 10.3390/ph14050448 (PMC8151064; doi:10.3390/ph14050448)
Supplement: Supplementary file 1 [file pharmaceuticals-14-00448-s001.zip › pharmaceuticals-1193210-supplementary.pdf]

SUPPLEMENTAL

Supplemental Table S1: Preparation of [^{68}Ga]Ga-NOTA-sdAb using ascorbic acid buffer system.

Compound	Buffer	Method	RCP (%)
NOTA-anti-HER2			10 min
	1M NaAcetate pH5	iTLC	92
		SEC	97
	0.5M NaAscorbate pH5	iTLC	1
		SEC	14

RCP = Radiochemical Purity; iTLC = instant Thin Layer Chromatography; SEC = Size Exclusion Chromatography
Mass precursor used per labeling = 100 µg

Supplemental Table S2: Verification study AA.

Compound	AA (mg/mL)	Incubation time AA	Method	RCP (%)
NOTA-anti-HER2				10 min
	5	Not verified	iTLC	68
	5	Not verified	iTLC	93
	5	Not verified	iTLC	84
	5	< 10 min	iTLC	96
	5	Overnight	iTLC	44

RCP = Radiochemical Purity; iTLC = instant Thin Layer Chromatography
Mass precursor used per labeling = 100 µg

Supplemental Table S3: Preparation of [^{68}Ga]Ga-NOTA-sdAb in combination with EtOH and PVP.

Compound	RP	Amount (mg/%)	Activity (GBq)	Method	QC (%)						pH
NOTA-anti-MMR lyo					10 min			3 h			
					**1**	**2**	**3**	**1**	**2**	**3**	
	PVP	100	1.10	iTLC	81	13	7	76	24	1	4.65
	PVP/EtOH	50/20	1.10	iTLC	83	5	12	82	12	6	4.53
	PVP/EtOH	50/40	1.07	iTLC	68	2	31	80	3	18	4.36

1 = RCP; 2 = radiolysis; 3 = unlabeled ^{68}Ga
RCP = Radiochemical Purity ; iTLC = instant Thin Layer Chromatography
Mass precursor used per labeling = 100 µg
The given concentration of PVP = concentration of PVP in the radiolabeling buffer
% EtOH = percentage ethanol in the labeling buffer, before addition of ^{68}Ga eluate

Supplemental Table S4 Preparation of [^{68}Ga]Ga-NOTA-sdAb in combination with EtOH and AA or GA.

Compound	RP	Amount ((mg/mL)/%)	Activity (MBq)	pH buffer	Activity Filter (%)	Method	RCP (%)	pH
NOTA-anti-MMR							10 min	
	/	/	458	5	3.3	iTLC	99	4.65
						SEC	99	
	AA/EtOH	5/20	489	5	2.0	iTLC	99	4.60
						SEC	/	
	AA/EtOH	1/20	476	5	2.2	iTLC	99	4.58
						SEC	100	
	GA/EtOH	5/20	470	5	2.1	iTLC	98	4.47
						SEC	99	
	GA/EtOH	1/20	459	5	2.0	iTLC	99	4.56
						SEC	99	

RCP = Radiochemical Purity; iTLC = instant Thin Layer Chromatography; SEC = Size Exclusion Chromatography
Mass precursor used per labeling = 200 μg
The given concentration of AA/GA = concentration of AA/GA in the radiolabeling buffer
% EtOH = percentage ethanol in the labeling buffer, before addition of ^{68}Ga eluate

Effect of EtOH on sdAb aggregation via SDS-PAGE

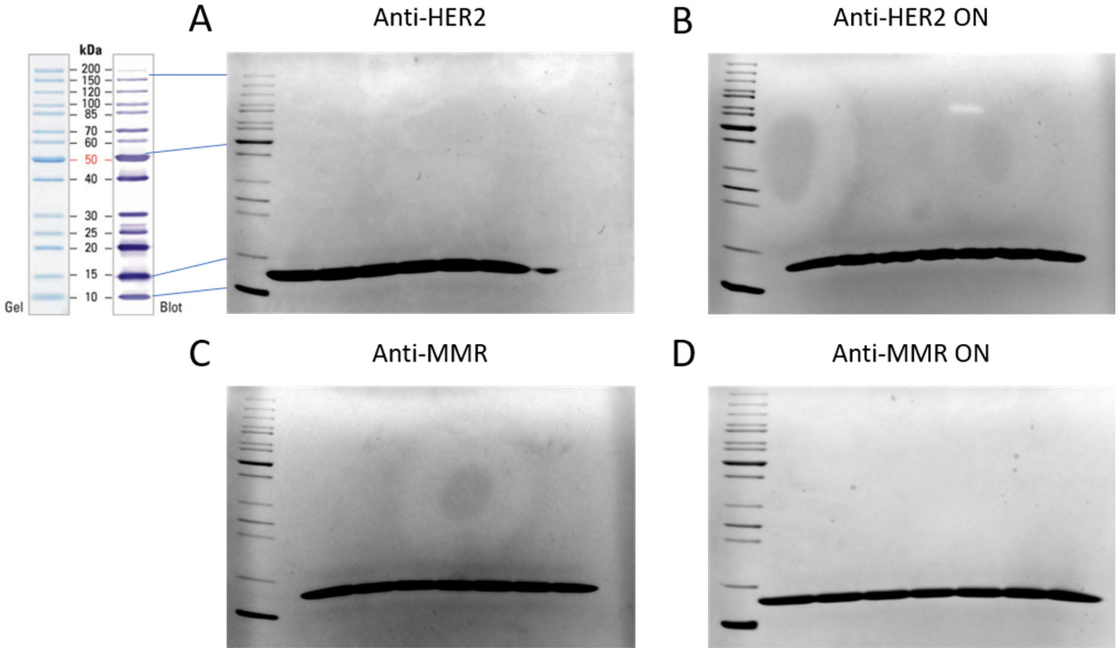

Supplemental Figure S1: Effect of EtOH on protein aggregation via SDS-PAGE. Protein were exposed to an increasing amount of EtOH, from 0–60 from left to right. (A) Analysis of anti-HER2 protein exposed to EtOH and analyzed withing 30 min, (B) Analysis of anti-HER2 exposed to EtOH and incubated overnight at 2–8°, (C) Analysis of anti-MMR protein exposed to EtOH and analyzed withing 30 min, (D) Analysis of anti-MMR exposed to EtOH and incubated overnight at 2–8°.

Particle Size Analysis via Dynamic Light Scattering

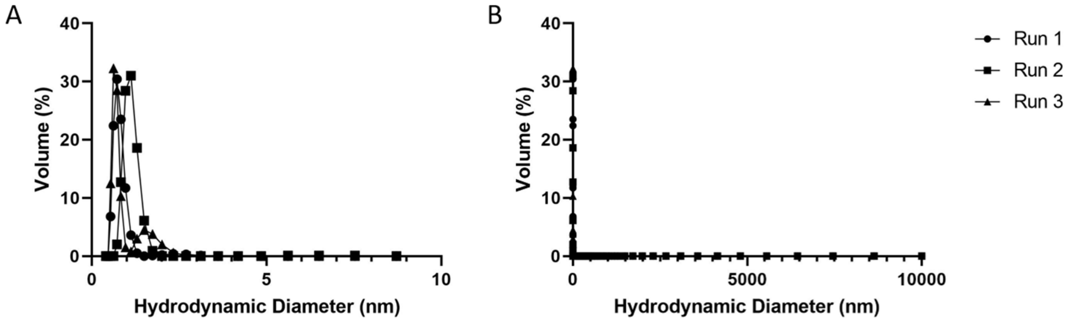

Supplemental Figure S2: Graphical representation of the distribution of sized particles in the final concentrated formulation. Panel 2A shows that the majority of particles has a maximum hydrodynamic diameter of 3 nm. Panel 2B shows the complete measurement spectrum and confirms that no other, larger, particle sizes are present in the solution.

Radio iTLC/SEC

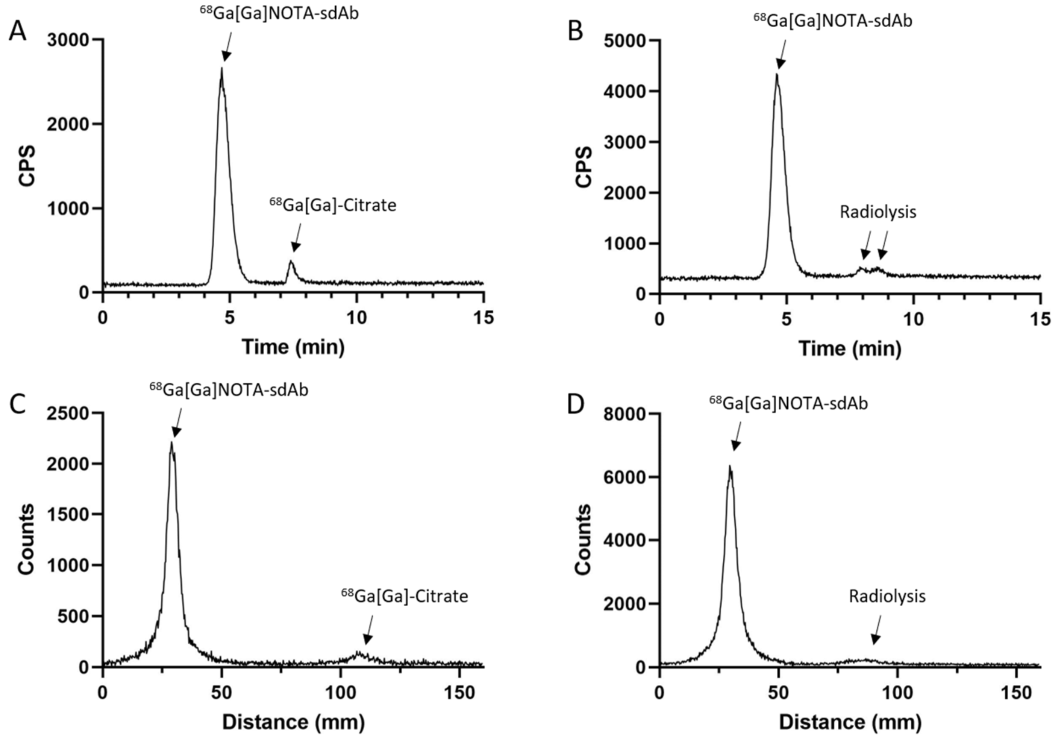

Supplemental Figure S3: Chromatographic analysis of ^{68}Ga[Ga]NOTA-sdAb, RCP and radiolysis. Panel A shows SEC analysis of ^{68}Ga[Ga]-NOTA-sdAb (R_t: 4.65 min) and non-incorporated ^{68}Ga[Ga]citrate (R_t: 7.40 min), while Panel B shows ^{68}Ga[Ga]-NOTA-sdAb (R_t: 4.60 min) with radiolytic degradation (R_t: 7.90 min—8.57 min). Panel C and D show the corresponding iTLC analysis, where Panel C shows ^{68}Ga[Ga]-NOTA-sdAb (R_f: 0.04 (28.7 mm)) and non-incorporated ^{68}Ga[Ga]citrate (R_f: 0.98 (107.8 mm)), while Panel D shows ^{68}Ga[Ga]-NOTA-sdAb (R_f: 0.07 (30.0 mm)) and radiolytic degration (R_f: 0.73 (87.1 mm)).
